# Ethnic Differences in Associations Between Fat Deposition and Incident Diabetes and Underlying Mechanisms: The SABRE Study

**DOI:** 10.1002/oby.20997

**Published:** 2015-02-03

**Authors:** Sophie V Eastwood, Therese Tillin, Hakim-Moulay Dehbi, Andrew Wright, Nita G Forouhi, Ian Godsland, Peter Whincup, Naveed Sattar, Alun D Hughes, Nishi Chaturvedi

**Affiliations:** 1 Institute of Cardiovascular Science, University College LondonUK; 2 Department of Epidemiology and Biostatistics, School of Public Health, Imperial College LondonLondon, UK; 3 Department of Medicine, Imperial College Healthcare NHS TrustLondon, UK; 4 MRC Epidemiology Unit, University of CambridgeUK; 5 Department of Endocrinology and Metabolic Medicine, Imperial College LondonUK; 6 Division of Population Health Sciences and Education, St. George's University of LondonUK; 7 Institute of Cardiovascular and Medical Sciences, University of Glasgow School of MedicineUK.

## Abstract

**Objective:**

To examine ethnic differences in ectopic fat and associations with incident diabetes.

**Methods:**

In a UK cohort study, 1338 Europeans, 838 South Asians, and 330 African Caribbeans living in London were aged 40-69 years at baseline. Baseline assessment included blood tests, anthropometry, and questionnaires. Anthropometry-based prediction equations estimated baseline visceral adipose tissue (VAT). Incident diabetes was ascertained from record review, self-report, or oral glucose tolerance testing.

**Results:**

South Asians had more and African Caribbeans less estimated VAT than Europeans. Both ethnic minorities had larger truncal skinfolds than Europeans. In men, adjustment for risk factors (BMI, smoking, systolic blood pressure, and HDL-cholesterol) markedly attenuated the association between estimated VAT and diabetes in Europeans (standardized subhazard ratios [95% CI]: from 1.74 [1.49, 2.03] to 1.16 [0.77, 1.76]) and African Caribbeans (1.72 [1.26, 2.35] to 1.44 [0.69, 3.02]) but not South Asians (1.60 [1.38, 1.86] to 1.90 [1.37, 2.64]). In women, attenuation was observed only for South Asians (1.80 [1.01, 3.23] to 1.07 [0.49, 2.31]). Associations between truncal skinfolds and diabetes appeared less affected by multivariable adjustment in South Asians and African Caribbeans than Europeans (1.24 [0.97, 1.57] and 1.28 [0.89, 1.82] versus 1.02 [0.77, 1.36] in men; 1.91 [1.03, 3.56] and 1.42 [0.86, 2.34] versus 1.23 [0.74, 2.05] in women).

**Conclusions:**

Differences in overall truncal fat, as well as VAT, may contribute to the excess of diabetes in South Asian and African Caribbean groups, particularly for women.

## Introduction

Burgeoning levels of obesity have led to a marked global rise in type 2 diabetes (1). Central obesity, in particular abdominal visceral adipose tissue (VAT), is thought to contribute to diabetes risk beyond general adiposity (2). Other ectopic fat depots may also increase diabetes risk, while abdominal and lower body subcutaneous adipose tissue (SAT) appears to be metabolically protective (3–5).

Migrant populations of South Asian and African Caribbean descent are at greater risk of type 2 diabetes than those of European descent, and this may relate to ethnic differences in body composition (4,6). In most studies, South Asians have more VAT than Europeans (7,8), whereas African Caribbeans have less (9,10); therefore abdominal obesity is an unlikely explanation for the excess diabetes seen in the latter group. We have previously identified truncal skinfold thickness as an independent risk factor for diabetes in South Asians and African Caribbeans (4), but it is unclear how this compares with VAT or SAT. Additionally, whilst lower body adiposity may have a favorable relationship with metabolic risk factors in Europeans (5), there is little research examining its influence in South Asians.

Many studies report longitudinal associations between waist circumference, waist/hip ratio, or BMI and incident diabetes (11). Few prospective studies have examined the impact of VAT and SAT on incident diabetes (12–15), and none that we are aware of have presented ethnic differences or included South Asians.

Using data from a community-based follow-up study, we assessed ethnic differences in estimated VAT, estimated SAT, and measures of overall truncal and lower body adiposity. Furthermore, we examined associations between baseline adiposity measures and incident diabetes, alone and in combination, by ethnicity.

## Methods

### Ethics statement

Participants gave written informed consent. Approval for the baseline study was obtained from Ealing, Hounslow and Spelthorne, Parkside, and University College London research ethics committees, and at follow-up from St. Mary's Hospital Local Research Ethics Committee (reference 07/HO712/109).

### Study sample

The SABRE study is a multiethnic cohort study of cardiometabolic disease; with details published elsewhere (16). Participants aged 40-69 years at baseline (1988-1991) were randomly selected from age- and sex-stratified primary care physician lists (*n* = 4063) and workplaces (*n* = 795) in north-west London. From this point, the terms “European,” “South Asian,” and “African Caribbean” refer to participants from those ethnic groups living in London. South Asian and African Caribbean participants were first-generation migrants—South Asians from the Indian subcontinent, (52% were of Punjabi Sikh origin) and African Caribbeans from the Caribbean (93%) or West Africa. Participants were followed up between 2008 and 2011, aged 58 to 85 years (*n* = 4196). The diabetes status of 2533 individuals was available at follow-up (Figure S1, Supporting Information), and 1410 of these attended a research clinic at follow-up. A male preponderance in the data exists in South Asians and Europeans as the baseline study was initially designed to examine cardiometabolic disease in men, but the distribution of men and women was more equal for African Caribbeans, who were recruited later into the study.

### Baseline measurements

Participants underwent fasting blood tests, blood pressure measurement and anthropometry, and completed a health and lifestyle questionnaire. Truncal skinfolds comprised the sum of sub-scapular and supra-iliac skinfolds, and leg skinfolds the sum of thigh and supra-patellar skinfolds. Anthropometry was measured by four trained observers, one acted as the standard and periodic standardization measurements were made during the study to ensure good interobserver reliability. Those whose diabetes status was unknown underwent oral glucose tolerance testing (OGTT). HOMA2-IR (17) and the Matsuda index (18) were used to quantify insulin resistance. Physician diagnosis (from self-report or record review) or World Health Organization 1999 criteria (19) for fasting and OGTT blood glucose measurements defined baseline diabetes. Physical activity comprised the total weekly energy expended (MJ) on sports, walking and daily activities, using questions based on the Allied Dunbar fitness survey (20) and energy expenditure estimates (21).

### Follow-up measurements

During 2008-2011, survivors participated in follow-up, including a health questionnaire, primary care medical record review, and/or attendance at clinic at St. Mary's hospital, London, where blood tests and anthropometry were performed. Additionally, VAT and SAT were measured by abdominal computer tomography (CT) scan at 125kVwith a Philips MX 8000 IDT64 detector, as previously described (7).

### Estimation of baseline abdominal visceral and SAT

In a previously published analysis of our follow-up data, we derived and rigorously evaluated prediction equations of CT-measured VAT and SAT by sex and ethnic group, using age and anthropometric measures (7). Equations took the following form:

VAT (or SAT) = *β*_0_+ (*β*_1_ × age) + (*β*_2_ × weight) + (*β*_3_ × height) + (*β*_4_ × waist circumference) + (*β*_5_ × hip circumference) + (*β*_6_ × thigh circumference)

Full equations are provided in Supporting Information Table S1. To estimate baseline VAT and SAT, we applied the appropriate sex- and ethnic-specific prediction equation to baseline anthropometric data.

### Identification of incident diabetes

Incident diabetes was identified from one of the following direct sources: primary care medical record review (recorded diagnosis of diabetes or prescription of antidiabetic medications), participant questionnaire (recall of physician-diagnosed diabetes plus either year of diagnosis or receipt of named antidiabetic medication), or follow-up at 20 years [fasting or OGTT plasma glucose results meeting World Health Organization 1999 criteria (19)]. Participants were also indirectly followed-up for diabetes, using death certificate data (diabetes listed as an underlying cause of death: ICD9 codes: 2500-2509, ICD 10 codes: E100-E149).

### Statistical analyses

Baseline characteristics were examined by follow-up status then compared between ethnic groups, stratified by sex and adjusted for age, using ANOVA or logistic regression models, as appropriate. Correlations between adiposity measures were inspected using Pearson's correlation coefficients, and ethnic differences tested for using Fisher's *z* transform test (22). We examined associations between depot-specific abdominal adiposity (estimated VAT and SAT), truncal skinfolds (as a validated marker of truncal subcutaneous fat), and leg skinfolds (as a marker of lower body subcutaneous fat) and diabetes, rather than general measures of adiposity, to elucidate the explicit effects of these variables and their inter-relationships. Informative censoring may have occurred due to death from causes other than diabetes; we addressed this by using competing risks regression (competing risk = death from nondiabetes cause), based on Fine and Grey proportional subhazards methods (23).

When assessing associations between estimated VAT or SAT and diabetes, we attempted to incorporate the uncertainty in the coefficients of the prediction equations used to obtain baseline estimated VAT and SAT values. Firstly, using follow-up data, we employed Bayesian linear regression models to estimate β-coefficients for the VAT and SAT prediction equations. Then the uncertainty in these estimates was propagated to the estimation of VAT and SAT at baseline. Finally, Monte-Carlo simulation (24) was used to perform the competing risk regressions evaluating the effect of estimated baseline VAT and SAT, adjusted for age, on incident diabetes, accounting for the uncertainty in baseline estimated SAT and VAT. Convergence was assessed using Gelman-Rubin diagnostics (24), and estimates based on 1500 samples after convergence.

We considered each adiposity measure in turn, and compared estimates adjusted for age (model 1) with those adjusted for age and BMI (model 2) to establish whether associations were independent of overall body size. Subsequently, we adjusted model 2 (adiposity measure + age + BMI) for the baseline cardiometabolic risk factors most associated with incident diabetes (smoking status, systolic blood pressure, and HDL-cholesterol), to establish whether associations were independent of these potential confounders (model 3). Following this, model 2 (adiposity measure + age + BMI) was then adjusted for baseline insulin resistance (measured by HOMA2-IR) (model 4), the Matsuda index, fasting glucose and fasting insulin (data not shown for latter 3 models) in turn. These analyses investigated potential mediating effects of insulin resistance on associations between adiposity and diabetes.

Following this, we studied age-adjusted competing risks models containing all body composition measures to examine their independent effects on diabetes, by sex, and ethnicity. Collinearity was assessed using variance inflation factors (VIFs), with a (VIF) >10.0 indicating model instability (25).

Interactions between the effects of ethnicity or sex and each body composition variable on diabetes were sought in all models. We examined Nelson-Aalen cumulative hazard plots by tertile of body composition variables to check for violations of the proportional hazards assumption; none were found. To test for bias due to case ascertainment method, we used logistic regression models to determine associations between body composition variables and diabetes where the date of diabetes diagnosis was unknown. Lastly, we further adjusted the maximally adjusted competing risks models for baseline heart rate and physical activity, as proxies for physical fitness. Analyses were performed in Stata 12 (College Station, Texas), using a statistical significance level of *P* < 0.05.

## Results

At baseline, 4202 individuals did not have diabetes, of whom 3908 (93%) were traced at follow-up to a UK address (Figure S1, Supporting Information). A total of 1338 (65%) Europeans, 838 (64%) South Asians, and 330 (61%) African Caribbeans had follow-up data and baseline body composition measurements available. With the exception of leg skinfolds, which were greater in those without follow-up data, baseline anthropometry, ethnicity, and metabolic parameters did not differ in participants with and without follow-up data (Table S2, Supporting Information). Over a median 19 years follow-up, incident type 2 diabetes was higher in South Asians and African Caribbeans than Europeans [34% (*n* = 281, *P* < 0.001) and 29% (*n* = 96, *P* < 0.001) vs. 14% (*n* = 191)].

South Asian men but not women had greater baseline estimated abdominal VAT than Europeans (Table1). However, South Asian women had greater estimated abdominal SAT and leg skinfolds than European women, in contrast to men where there was no ethnic difference. Of note, South Asians of both sexes had larger truncal skinfolds than Europeans. African Caribbean men and women had less estimated VAT and smaller leg skinfolds than Europeans (Table1). African Caribbean men had less, and women more, estimated SAT then Europeans. Truncal skinfolds were also larger in African Caribbean men and women, when compared with Europeans. Men and women of South Asian and African Caribbean origin generally had adverse blood pressure, lipid, glycemic, and insulin resistance profiles, compared with Europeans (Table1).

**Table 1:** Baseline characteristics: SABRE study

Variable	Europeans	South Asians	*P*^a^	African Caribbeans	*P*^a^
***In men without diabetes***					
***n***	1043	702	-	185	-
**Age (years)**	52 ± 7	51 ± 7	<0.0001	53 ± 6	0.08
**Estimated VAT (cm^2^)**	127 ± 74	161 ± 67	<0.0001	113 ± 66	0.001
**Estimated SAT (cm^2^)**	196 ± 62	193 ± 63	0.22	148 ± 67	<0.0001
**Truncal skinfolds (mm)**	39 ± 14	47 ± 14	<0.0001	43 ± 18	0.003
**Leg skinfolds (mm)**	22 ± 8	22 ± 8	0.71	19 ± 7	<0.0001
**Waist circumference (cm)**	91 ± 10	92 ± 10	0.01	89 ± 10	0.001
**BMI (kg/m^2^)**	26 ± 4	25 ± 3	0.03	26 ± 3	0.46
**Ever smoked (%)**	72	26	<0.001	44	<0.001
**Heart rate (beats/min)**	65 ± 11	67 ± 10	<0.0001	63 ± 10	0.02
**Physical activity (MJ/wk)**	11 (7-16)	10 (6-13)	<0.0001	10(7-15)	0.17
**Systolic BP (mmHg)**	121 ± 16	124 ± 17	0.001	127 ± 15	<0.0001
**HDL (mmol/l)**	1.3 ± 0.3	1.2 ± 0.3	<0.0001	1.5 ± 0.4	<0.0001
**Fasting glucose (mmol/l)**	5.4 ± 0.6	5.5 ± 0.6	<0.0001	5.5 ± 0.6	0.02
**Postload glucose (mmol/l)**	5.0 ± 1.3	5.4 ± 1.5	<0.0001	5.8 ± 1.6	<0.0001
**Fasting insulin (pmol/l)**	7(5-11)	10 (7-14)	<0.0001	8 (5-11)	0.31
**Postload insulin (pmol/l)**	20 (12-33)	40 (23-73)	<0.0001	25 (16-40)	0.001
**HbA_1c_ (%)**	5.5 (5.3-5.7)	5.7(5.5-6.0)	<0.0001	5.8 (5.5-6.0)	<0.0001
**HbA_1c_ (mmol/mol)**	37 (34-39)	39(37-42)	<0.0001	40 (37-42)	<0.0001
**HOMA2-IR**	0.8 (0.6-1.2)	1.1(0.8-1.6)	<0.0001	1.0 (0.6-1.3)	0.14
**Matsuda index**	0.11 (0.07-0.17)	0.18 (0.12-0.30)	<0.0001	0.14 (0.09-0.21)	0.0004
***In women without diabetes***					
***n***	295	136	-	145	-
**Age (years)**	53 ± 7	50 ± 6	<0.0001	52 ± 6	0.49
**Estimated VAT (cm^2^)**	85 ± 57	86 ± 46	0.35	77 ± 50	0.36
**Estimated SAT (cm^2^)**	248 ± 88	323 ± 80	<0.0001	294 ± 113	<0.0001
**Truncal skinfolds (mm)**	40 ± 19	67 ± 15	<0.0001	53 ± 20	<0.0001
**Leg skinfolds (mm)**	53 ± 19	67 ± 16	<0.0001	48 ± 18	0.09
**BMI (kg/m^2^)**	26 ± 5	27 ± 4	0.02	29 ± 5	<0.0001
**Waist circumference (cm)**	79 ± 12	84 ± 10	0.0001	88 ± 12	<0.0001
**Ever smoked (%)**	52	3	<0.001	19	<0.001
**Heart rate (beats/min)**	65 ± 9	70 ± 11	<0.0001	64 ± 11	0.14
**Physical activity (MJ/wk)**	8 (5-13)	6 (1-10)	<0.0001	10 (7-13)	0.02
**Systolic BP (mmHg)**	119 ± 17	123 ± 22	<0.0001	129 ± 16	<0.0001
**HDL (mmol/l)**	1.7 ± 0.5	1.4 ± 0.3	<0.0001	1.7 ± 0.4	0.86
**Fasting glucose (mmol/l)**	5.3 ± 0.5	5.0 ± 0.5	<0.0001	5.4 ± 0.6	0.05
**Postload glucose (mmol/l)**	5.8 ± 1.4	5.7 ± 1.2	0.55	6.5 ± 1.5	<0.0001
**Fasting insulin (pmol/l)**	5 (4-8)	7 (5-10)	<0.0001	10 (6-13)	<0.0001
**Postload insulin (pmol/l)**	22 (15-35)	44 (26-67)	<0.0001	36 (24-57)	<0.0001
**HbA_1c_ (%)**	5.4 (5.2-5.7)	5.7 (5.4-6.0)	0.0001	5.7 (5.4-6.2)	0.26
**HbA_1c_ (mmol/mol)**	36 (33-39)	39 (36-42)	0.0001	39 (36-424)	0.26
**HOMA2-IR**	0.6 (0.4-0.9)	0.8 (0.6-1.1)	0.002	1.1 (0.7-1.4)	<0.0001
**Matsuda index**	0.10 (0.07, 0.16)	0.17 (0.10-0.24)	<0.0001	0.19 (0.13-0.29)	<0.0001

Data are mean ± SD or median (IQR) unless otherwise indicated.

a Age-adjusted *P* for ethnic difference, when compared with Europeans.

VAT: visceral adipose tissue; SAT: subcutaneous adipose tissue; truncal skinfolds: subscapular skinfold + suprailiac skinfold; leg skinfolds: thigh skinfold + suprapatellar skinfold; BP: blood pressure.

Estimated VAT was highly correlated with estimated SAT in univariate analyses, but less so with leg skinfolds (Table S3, Supporting Information). There were no consistent ethnic differences in correlations. In general, higher adiposity measures were adversely associated with incident diabetes in age-adjusted models (Table2). There were no statistically significant sex or ethnic differences in the effects of adiposity measures. In these models, the measures most associated with incident diabetes were estimated SAT in European and African Caribbean men, estimated VAT in South Asian men, European women and African Caribbean women, and truncal skinfolds in South Asian women. Additionally, we studied age-adjusted models where uncertainty in the VAT and SAT prediction equations had been accounted for within a Bayesian framework. These showed estimates with similar directions, magnitudes, margins of uncertainty, and interethnic patterns as the main univariate models (Figures S2 and S3, Supporting Information).

**Table 2:** Multivariable associations between adiposity measures and incident diabetes: SABRE study

Adiposity measure	Model	Europeans		South Asians		African Caribbeans	
		SHR	95% CI	SHR	95% CI	SHR	95% CI
***In men***							
**VAT**	1	1.74***	1.49, 2.03	1.60***	1.38, 1.86	1.72**	1.26, 2.35
	2	1.43*	1.00, 2.05	2.18***	1.61, 2.96	1.38	0.71, 2.66
	3	1.16	0.77, 1.76	1.90***	1.37, 2.64	1.44	0.69, 3.02
	4	1.32	0.90, 1.94	1.87***	1.36, 2.55	1.35	0.70, 2.62
**SAT**	1	2.05***	1.67, 2.52	1.59***	1.36, 1.87	1.74**	1.23, 2.46
	2	1.22	0.51, 2.93	2.81**	1.46, 5.42	1.03	0.39, 2.64
	3	0.74	0.27, 2.04	2.67**	1.37, 5.23	1.25	0.48, 3.22
	4	0.84	0.35, 2.03	2.21*	1.11, 4.37	1.04	0.41, 2.68
**Truncal skinfolds**	1	1.73***	1.46, 2.07	1.49***	1.29, 1.72	1.57***	1.24, 1.98
	2	1.21	0.94, 1.56	1.21	0.99, 1.49	1.34	0.96, 1.86
	3	1.02	0.77, 1.36	1.24	0.97, 1.57	1.28	0.89, 1.86
	4	1.11	0.86, 1.43	1.07	0.86, 1.33	1.27	0.89, 1.82
**Leg skinfolds**	1	1.87***	1.38, 2.54	1.60**	1.22, 2.11	1.13	0.54, 2.37
	2	1.06	0.71, 1.59	1.03	0.73, 1.47	0.42	0.15, 1.22
	3	1.00	0.62, 1.60	1.08	0.72, 1.62	0.49	0.17. 1.41
	4	1.04	0.68, 1.58	0.87	0.61, 1.25	0.41	0.13, 1.19
***In women***							
**VAT**	1	2.35***	1.74, 3.19	1.80*	1.01, 3.23	2.85***	1.84, 4.41
	2	2.66*	1.14, 6.20	1.24	0.59, 2.58	3.36**	1.50, 7.53
	3	2.71*	1.16, 6.34	1.07	0.49, 2.31	3.18**	1.37, 7.39
	4	2.39	0.97, 5.89	0.96	0.45, 2.04	3.32*	1.21, 9.11
**SAT**	1	1.70***	1.37, 2.12	1.44	0.98, 2.12	1.29*	1.05, 1.59
	2	1.08	0.48, 2.46	0.82	0.26, 2.60	0.79	0.44, 1.43
	3	0.93	0.44, 1.96	0.94	0.29, 3.07	0.84	0.47, 1.51
	4	1.09	0.55, 2.18	0.90	0.28, 2.89	0.83	0.44, 1.56
**Truncal skinfolds**	1	1.82***	1.38, 2.38	2.00**	1.27, 3.13	1.70***	1.29, 2.23
	2	1.43	0.93, 2.20	1.91*	1.05, 3.48	1.65*	1.07, 2.54
	3	1.23	0.74, 2.05	1.91*	1.03, 3.56	1.42	0.86, 2.34
	4	1.37	0.89, 2.11	1.93*	1.05, 3.55	1.44	0.89, 2.32
**Leg skinfolds**	1	1.68**	1.15, 2.44	1.59*	1.04, 2.43	1.39	0.98, 1.97
	2	1.19	0.80, 1.77	1.36	0.76, 2.43	1.07	0.59, 1.95
	3	1.07	0.69, 1.65	1.41	0.80, 2.45	1.19	0.64, 2.19
	4	1.23	0.84, 1.82	1.38	0.79, 2.43	1.20	0.64, 2.27

SHR: subhazard ratio (competing risks models) showing the effect of a 1 SD increase in each adiposity measure on incident diabetes.

* *P* < 0.05

** *P* < 0.01

*** *P* < 0.001. Model 1: adjusted for age, model 2: adjusted for age + BMI, model 3: adjusted for age + BMI + smoking status + systolic blood pressure + HDL-cholesterol, model 4: adjusted for age + BMI + HOMA2-IR.

VAT: visceral adipose tissue; SAT: subcutaneous adipose tissue.

We inspected models of age and BMI-adjusted associations between each adiposity measure and diabetes (Table2). This base model was then further adjusted for cardiometabolic risk factors and insulin resistance in turn. In European men, estimated VAT was the only measure to retain a strong association with diabetes after BMI adjustment; this was attenuated by adjustment for cardiometabolic risk factors and, to a lesser extent, for insulin resistance. In South Asian men, associations between estimated VAT and diabetes increased after adjustment for BMI, and remained (although reduced) when further adjusted for cardiometabolic risk factors and insulin resistance. Collinearity diagnostics showed a VIF of >10 for estimated SAT and BMI in South Asian men, so these results should be interpreted with caution. BMI-adjusted associations between estimated VAT and diabetes appeared stronger in South Asian than European men [subhazard ratios (SHR): 2.18, (95% CI;1.61, 2.96), versus 1.43 (1.00,2.05)], although this was not significant as an interaction (*P* = 0.47). After BMI adjustment, nonsignificant adverse associations with diabetes persisted for estimated VAT and for truncal skinfolds in African Caribbean men [SHRs (95% CI):1.38 (0.71, 2.66), *P* = 0.34 and 1.36, (0.96, 1.86), *P* = 0.08, respectively]. Additionally, there appeared to be a weak protective association for leg skinfolds; none of these associations were greatly affected by further adjustment.

In European women, strong associations persisted for estimated VAT after BMI adjustment, which were diminished in the insulin resistance model. Truncal skinfolds were still associated with diabetes independent of BMI in South Asian women, and neither cardiometabolic risk factors nor insulin resistance could account for the association with diabetes. In African Caribbean women, associations persisted for estimated VAT and truncal skinfolds independent of BMI; after accounting for cardiometabolic risk factors or insulin resistance, these associations remained largely unaltered.

**Figure 1:**
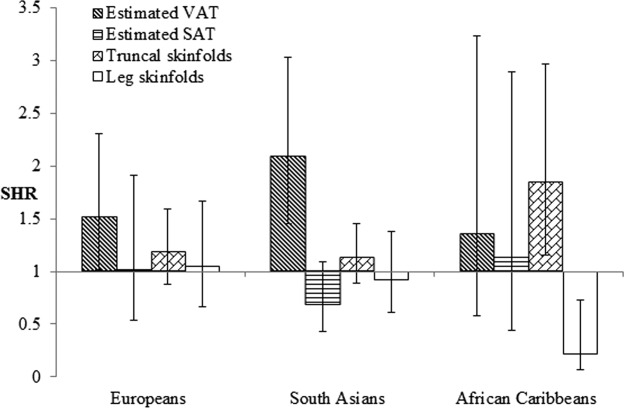
Multivariable associations between adiposity measures considered in combination and incident diabetes in men: SABRE study. SHR: subhazard ratio (competing risks models) showing the effect of a 1 SD increase in each adiposity measure on incident diabetes; lines indicate 95% confidence intervals (CI). Model factors comprised age, estimated VAT, estimated SAT, truncal skinfolds, and leg skinfolds. SAT: subcutaneous adipose tissue; VAT: visceral adipose tissue.

**Figure 2:**
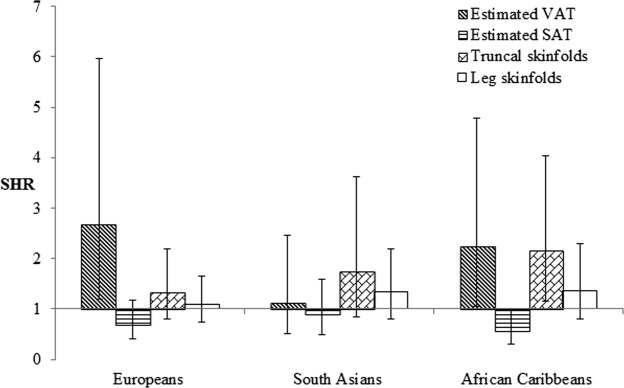
Multivariable associations between adiposity measures considered in combination and incident diabetes in women: SABRE study. SHR= subhazard ratio (competing risks models) showing the effect of a 1 SD increase in each adiposity measure on incident diabetes; lines indicate 95% confidence intervals (CI). Model factors comprised age, estimated VAT, estimated SAT, truncal skinfolds and leg skinfolds. SAT: subcutaneous adipose tissue; VAT: visceral adipose tissue.

We examined multivariable models of all adiposity measures of interest in combination (estimated VAT and SAT, truncal, and leg skinfolds; Figures 1 and 2). Only estimated VAT retained independent associations with diabetes in European and South Asian men, whilst in African Caribbean men, detrimental associations persisted only for truncal skinfolds, and leg skinfolds appeared to be protectively associated. Associations between estimated VAT and diabetes were greater in South Asian then European men [SHR: 2.09, (95% CI; 1.45, 3.03) vs. 1.52 (1.02, 2.30)], but again this was not significant as an interaction (*P* = 0.76). In European and African Caribbean women, estimated VAT remained adversely associated with diabetes, and truncal skinfolds were also unfavorably associated in the latter group. In South Asian women, only truncal skinfolds appeared independently associated, albeit nonsignificantly (SHR: 1.74, 95% CI: 0.84, 3.62, *P* = 0.14). Patterns of associations were identical when these models were further adjusted for BMI.

With the exception of the BMI-adjusted SAT models for South Asian men, collinearity was within acceptable limits for all models. When multivariable models were adjusted for the Matsuda index, fasting glucose and fasting insulin, results were similar in all cases to those seen with adjustment for HOMA2-IR, though the latter metric had the most consistent impact on the associations between adiposity and diabetes. No statistically significant interactions between sex or ethnicity and adiposity measures were observed in multivariable models. Analyses including individuals with no date of diabetes diagnosis produced identical patterns to the main analyses, as did those adjusting for baseline heart rate and physical activity levels (data not shown).

## Discussion

There were distinct patterns of fat distribution by ethnicity: South Asians and African Caribbeans had larger truncal skinfolds than Europeans. South Asian men had more estimated VAT than Europeans, while African Caribbeans of both sexes had less. Accounting for BMI, truncal adiposity was most strongly associated with diabetes in South Asian women, whereas estimated VAT was most strongly associated for all other groups. Associations between estimated VAT and diabetes appeared stronger in South Asian than European men, and in women than men of all ethnic groups. Adjustment for insulin resistance attenuated associations between all fat depots and diabetes in European men; patterns were not consistent in the other groups, with the exception of South Asian men, in whom the effects were retained.

Our findings of greater estimated VAT in South Asians than Europeans match previous studies (8). Furthermore, comparisons of South Asian and European children show that by early childhood, South Asians have greater truncal fat deposition (26), persisting into adulthood (27). This may arise due to a hyperinsulinemic in utero environment, micronutrient deficiencies, or genetic differences (26). Little data exist comparing lower body adiposity in these groups; however, a recent study showed larger leg skinfolds in South Asians than in Europeans (28), which we found only in women. Previous studies comparing African and European origin populations have also shown less VAT and larger truncal skinfolds in the African origin groups (9,10,29). Studies comparing leg adiposity in these ethnic groups show inconsistent results (30,31).

The independent associations between estimated VAT and incident diabetes we found in Europeans, South Asian men, and African Caribbean women reflect previous findings (12–15). Our results suggest the influence of VAT on the development of diabetes may be greater in South Asian than European men. Although we are not aware of any longitudinal studies comparing the effects of VAT in these ethnic groups, cross-sectional work relating VAT to insulin resistance hints this may be the case (8). Thus the excess diabetes risk in South Asian men may be explained by both their greater amount of VAT and its more adverse effects. Explanations for the more deleterious role of VAT in South Asian than European men may relate to differences in adipocyte morphology (32). There was also a indication of a greater association between estimated VAT and diabetes in women than men, reflecting findings from one longitudinal study (14), but not others (12,13,15); explanations are unclear.

We are not aware of any longitudinal studies showing that truncal skinfolds are better associated with diabetes than VAT, as we found in South Asian women, though as far as we know, no study has examined these measures in combination (12–15). However, cross-sectional studies have implied this: in a comparison of VAT, SAT and truncal skinfolds, Abate et al. found that truncal skinfolds were most strongly related to insulin sensitivity (33). A possible explanation is that a higher proportion of circulating FFA, including those reaching the liver, are released from upper body subcutaneous rather than VAT (34). However, when there is relatively greater deposition of VAT than SAT, its role in hepatic insulin resistance may increase, although upper body SAT remains important in driving peripheral insulin resistance (35).

The balance of subcutaneous and visceral fat varies with ethnicity—the higher levels of estimated VAT and overall truncal subcutaneous fat seen in South Asians when compared with Europeans may point to a propensity for both central (VAT-mediated) and peripheral (SAT-mediated) insulin resistance. However, the higher levels of truncal subcutaneous fat seen in African Caribbeans suggest a tendency toward peripheral insulin resistance, which may contribute to their excess diabetes risk in the absence of high levels of estimated VAT. Yet in general, baseline differences in insulin resistance did not fully explain associations between either fat depot and diabetes in this study. This may be due to imprecision in our estimates of insulin resistance. Alternatively, the interplay between adiposity, insulin resistance, and subsequent diabetes may be moderated by factors which we were unable to explore, for example, inflammation.

In our age-adjusted analyses, estimated SAT and leg skinfolds appeared to be adversely associated with diabetes, probably as a marker of overall adiposity, since these associations were attenuated or reversed in most groups once other fat depots were accounted for, as others have shown (5). Few studies report longitudinal associations between lower body adiposity and diabetes (and none that we know of by ethnicity), and those that do present contradictory results (36,37). The effects of leg adipose tissue may vary with ethnicity, and this is why we only found it to be negatively associated in African Caribbean men.

This study is novel in presenting longitudinal associations between specific fat depots and diabetes in three ethnic groups, and is the first such study in South Asians that we know of. Other strengths include a comparatively large sample size (12–15) and the focus on four adiposity measures, allowing a more nuanced picture of the interplay between upper and lower body adiposity (12–15). As with any cohort study, loss to follow-up may have introduced bias, though reasonable proportions of those traced were followed-up (61-65%), and an analysis comparing responders and nonresponders found no consistent differences in baseline cardiometabolic or adiposity variables. The latter finding implies follow-up data was missing at random, and thus the observed attrition rates are unlikely to introduce bias (38). Coupled with our use of random sampling of the study population at baseline, this suggests our findings may be generalizable to mid- to late-life populations of these three ethnic groups resident in the UK, although in some groups, numbers were small and thus the estimates presented in multivariable models have wide margins of uncertainty. The use of prediction equations to approximate baseline VAT and SAT will have resulted in a less precise estimate of these depots than direct measurement from CT or magnetic resonance (MR) imaging. However, the widespread use of CT or MR techniques for quantifying fat depots is a recent phenomenon, and thus no long term cohorts have employed these techniques. Furthermore, when longitudinal analyses were repeated in a Bayesian framework allowing for error in the prediction equations, associations remained similar. Moreover, we have shown that these models fitted our follow-up data reasonably well (adjusted *R*^2^ 0.55-0.86) and performed soundly in a cross-validation exercise (7).

In summary, South Asians and African Caribbeans had greater overall truncal adiposity, and South Asian men greater estimated VAT, than Europeans. Accounting for overall adiposity, estimated VAT was the strongest correlate of incident diabetes in most groups, although truncal fat showed the strongest associations in South Asian women. Associations were generally not explained by differences in cardiometabolic risk factors or insulin resistance. Further work should focus on defining the characteristics of upper and lower body fat in these groups, and exploring reasons for their expansion and adverse cardiometabolic effects.
