# Supplementary material for: Ethnic Differences in Associations Between Fat Deposition and Incident Diabetes and Underlying Mechanisms: The SABRE Study
Source: Obesity (Silver Spring). 2015 Feb 3;23(3):699–706. doi: 10.1002/oby.20997 (PMC4463764; doi:10.1002/oby.20997)
Supplement: Supplementary file 1 [file oby0023-0699-sd1.pdf]

Supporting information

Ethnic differences in associations between fat deposition and incident diabetes, and underlying mechanisms: the SABRE study.

Sophie V Eastwood, Therese Tillin, Hakim-Moulay Dehbi, Andrew Wright, Nita G Forouhi, Ian Godsland, Peter Whincup, Naveed Sattar, Alun D Hughes, Nishi Chaturvedi

Corresponding author: Dr Sophie V Eastwood, UCL Institute of Cardiovascular Sciences, UCL, Gower Street, London, United Kingdom, WC1E 6BT. Email: sophie.eastwood@ucl.ac.uk, Tel: +44 (0)207 679 9434.

Supporting information

a) **VAT in cm²**

Gender/ ethnic group	Prediction equation	Adjusted R^2
White European men	67.88 + (1.87 x age) + (2.80 x weight) – (1.79 x height) + (7.57 x waist) – (4.91 x hip) – (2.85 x thigh)	0.69
South Asian men	39.24 + (1.18 x age) + (2.16 x weight) – (2.35 x height) + (6.56 x waist) – (2.15 x hip) – (1.81 x thigh)	0.61
African Caribbean men	-422.31 + (0.94 x age) + (8.63 x waist) - (3.86 x hip) + (1.47 x thigh)	0.73
White European women	-250.86 + (0.90 x age) + (2.71 x weight) + (2.62 x waist) – (1.75 x thigh)	0.62
South Asian women	475.69 + (1.13 x age) + (6.28 x weight) – (2.47 x height) + (1.85 x waist) – (3.71 x hips) – (4.35 x thigh)	0.55
African Caribbean women	323.39 +(2.20 x age) + (3.20 x weight) – (2.42 x height) + (3.35 x waist) – (4.93 x hip)	0.56

b) **SAT in cm²**

Gender/ ethnic group	Prediction equation	Adjusted R^2
White European men	-28.95 – (1.19 x age) + (2.29 x weight) – (2.00 x height) + (2.34 x waist) + (2.49 x hip)	0.72
South Asian men	48.58 + (0.63 x age) + (3.88 x weight) – (3.21 x height) + (1.43 x waist) + (2.54 x hip)	0.75
African Caribbean men	-373.61 – (0.08 x age) – (2.09 x height) + (3.83 x waist) + (6.52 x hip) – (1.23 x thigh)	0.81
White European women	24.98 – (0.99 x age) + (3.76 x weight) – (3.07 x height) + (7.01 x hip) – (3.03 x thigh)	0.80
South Asian women	404.25 – (1.72 x age) + (3.94 x weight) – (5.21 x height) + (5.63 x hip)	0.81
African Caribbean women	-896.27 + (0.23 x age) + (2.24 x waist) + (9.48 x hip)	0.86

Table S1. Prediction equations for a) VAT and b) SAT, by sex and ethnicity: SABRE study (7). Models used the following predictors for both depots: age (years), weight (kg), height (cm), waist circumference (cm), hip circumference (cm), thigh circumference (cm).

Supporting information

Variable	Participants with available follow-up data	Participants without available follow-up data	p
Age, years	52.2±7.0	52.6±6.8	0.06
Non-European ethnicity, %	51	53	0.13
Estimated VAT, cm^2	133±76	133±76	0.98
Estimated SAT, cm^2	213±84	281±85	0.07
Truncal skinfolds, mm	45±18	45±18	0.30
Leg skinfolds, mm	29±17	31±19	<0.001
Ever smoked, %	47	46	0.63
Physical activity (MJ/wk)	10(6-14)	10(5-14)	0.27
Systolic BP, mmHg	124±18	125±17	0.06
HDL, mmol/l	1.30(1.08-1.57)	1.32(1.08-1.61)	0.05
Fasting glucose, mmol/l	6.02±2.18	5.90±1.92	0.28
Post-load glucose, mmol/l	6.07±2.98	5.95±2.64	0.15
Fasting insulin, pmol/l	8.40(5.31-12.50)	8.00(5.30-12.49)	0.19
Post-load insulin, pmol/l	27.80(16.40-49.30)	26.96(15.80-47.10)	0.14
HbA_{1c}, %	5.7(5.4-6.0)	5.7(5.4-6.0)	0.33
HOMA2-IR	1.0(0.6-1.5)	0.9(0.6-1.4)	0.13
Matsuda index	0.15(0.09-0.25)	0.14(0.09-0.24)	0.11

Table S2. Comparison of baseline characteristics for individuals with and without follow-up data for diabetes: SABRE study. Data are mean±SD or median (IQR) unless otherwise indicated, VAT= visceral adipose tissue, SAT= subcutaneous adipose tissue, truncal skinfolds= subscapular skinfold + suprailiac skinfold, leg skinfolds= thigh skinfold + suprapatellar skinfold.

Supporting information

European men

	VAT	SAT	TSF	LSF
**VAT**	1			
**SAT**	0.83*	1		
**TSF**	0.61*	0.74*	1	
**LSF**	0.34*	0.53*	0.62*	1

South Asian men

	VAT	SAT	TSF	LSF
**VAT**	1			
**SAT**	**0.91***	1		
**TSF**	0.56*	**0.65***	1	
**LSF**	0.44*	0.55*	0.65*	1

African Caribbean men

	VAT	SAT	TSF	LSF
**VAT**	1			
**SAT**	**0.91***	1		
**TSF**	**0.75***	0.77*	1	
**LSF**	**0.51***	0.55*	0.64*	1

European women

	VAT	SAT	TSF	LSF
**VAT**	1			
**SAT**	0.88*	1		
**TSF**	0.74*	0.72*	1	
**LSF**	0.48*	0.57*	0.69*	1

South Asian women

	VAT	SAT	TSF	LSF
**VAT**	1			
**SAT**	**0.51***	1		
**TSF**	**0.55***	0.66*	1	
**LSF**	0.31*	0.71*	0.55*	1

African Caribbean women

	VAT	SAT	TSF	LSF
**VAT**	1			
**SAT**	**0.51***	1		
**TSF**	0.64*	0.74*	1	
**LSF**	**0.17**	0.71*	0.60*	1

Table S3. Correlations between adiposity measures, by sex and ethnicity: SABRE study. Data are Pearson's correlation coefficients, *p<0.001. Coefficients shown in bold font were significantly different to those seen in Europeans (P<0.005). VAT= visceral adipose tissue, SAT= subcutaneous adipose tissue, TSF=truncal skinfolds= subscapular skinfold + suprailiac skinfold, LSF=leg skinfolds= thigh skinfold + suprapatellar skinfold.

Supporting information

	Europeans	South Asians	African Caribbeans
**Baseline, 1988-91**	n=2346	n=1710	n=801
**No baseline diabetes**	n=2197	n=1355	n=650
**Traced at follow-up, 2008-11**	n=2063	n=1300	n=545
**Follow up for diabetes**	n=1356	n=842	n=335
**All adiposity measures available**	n=1338 (65% traced)	n=838 (64% traced)	n=330 (61% traced)

Figure S1. Follow-up of the SABRE cohort, 1988-2011.

Supporting information

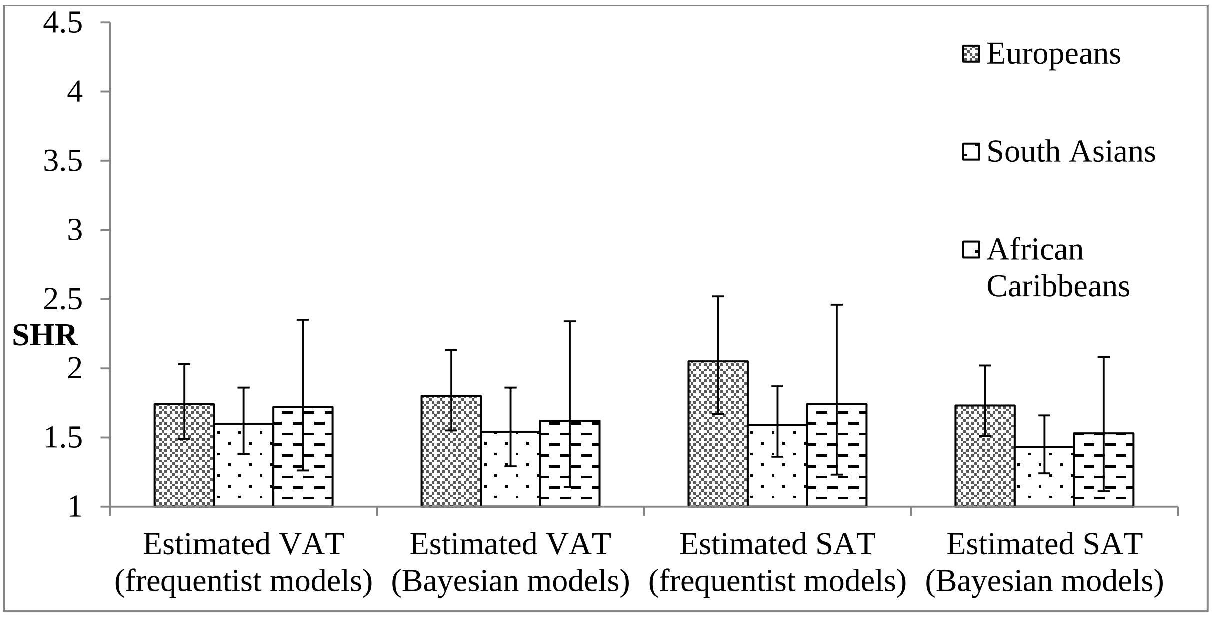

Figure S2. Age-adjusted univariate associations between estimated VAT or SAT and diabetes in men: a comparison of estimates with and without allowances for uncertainty in the coefficients of the prediction equations used to derive baseline estimated VAT or SAT. SHR=sub-hazard ratio (competing risks models) showing the effect of a 1 SD increase in each adiposity measure on incident diabetes, lines indicate 95% confidence intervals (frequentist models) or 95% credible intervals (Bayesian models). Bayesian models incorporate uncertainty in the beta-coefficients of the prediction equations used to derive baseline adiposity measures, whereas frequentist models do not. VAT= visceral adipose tissue, SAT= subcutaneous adipose tissue.

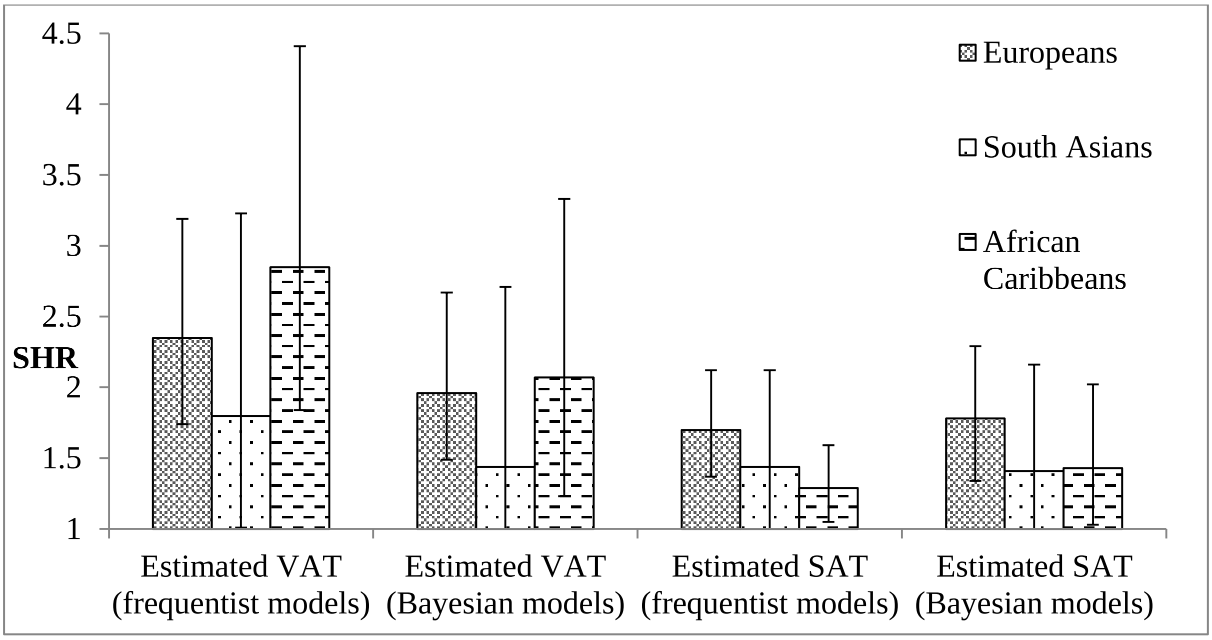

Figure S3. Age-adjusted univariate associations between estimated VAT or SAT and diabetes in women: a comparison of estimates with and without allowances for uncertainty in the coefficients of the prediction equations used to derive baseline estimated VAT or SAT. SHR=sub-hazard ratio (competing risks models) showing the effect of a 1 SD increase in each adiposity measure on incident diabetes, lines indicate 95% confidence intervals (frequentist models) or 95% credible intervals (Bayesian models). Bayesian models incorporate uncertainty in the beta-coefficients of the prediction equations used to derive baseline adiposity measures, whereas frequentist models do not. VAT= visceral adipose tissue, SAT= subcutaneous adipose tissue.
